# Pheasants 365: A Review of Seasonal Ring‐Necked Pheasant Habitat in Central North America

**DOI:** 10.1002/ece3.71867

**Published:** 2025-07-30

**Authors:** Allison J. Barg, Megan Baldissara, Daniel R. Uden, John P. Carroll, Andrew Little

**Affiliations:** ^1^ School of Natural Resources University of Nebraska‐Lincoln Lincoln Nebraska USA; ^2^ School of Natural Resources, Department of Agronomy and Horticulture, Center for Resilience in Agricultural Working Landscapes University of Nebraska‐Lincoln Lincoln Nebraska USA

**Keywords:** agricultural landscapes, avian ecology, game bird, grassland conservation, habitat management, *Phasianus colcichus*

## Abstract

Ring‐necked pheasants, an economically important game species in North America, are facing population declines in agriculturally dominated areas due to the intensification of production systems. There is a significant body of research on pheasant habitat requirements, much of which aims to address declines by guiding management decisions to improve habitat conditions. Pheasants, like many species, have differing resource needs throughout the year based on their life history and seasonal changes to the landscape; however, many studies have only focused on one or two of the three key ecological seasons—breeding, brood‐rearing, or overwintering. We conducted a literature review using the Web of Science database to synthesize results from studies investigating pheasant habitat in central North America in a seasonal context in order to provide a year‐round perspective on pheasant habitat management. Our results show the importance of grasslands and small grains during the nesting and brood‐rearing seasons, while wetlands and food plots become important during the winter months. Grassland structure is a crucial component of nesting habitat, with pheasants selecting for greater litter content and visual obstruction for nesting in 90% of studies that included those variables, while during brood‐rearing, chicks had higher success in grasslands with a higher percentage of forbs interspersed with bare ground. No consistent relationship was found with woodlands during any season, positive or negative. Pheasant relationships with row crops showed mixed results during nesting and brood‐rearing, as they are frequently used but not preferred. During the winter, row crops and other agricultural lands such as pasture and hay fields were negatively related to pheasant resource use, abundance, and/or survival. This review aims to guide management decisions throughout the entire year for pheasant habitat by providing a season‐specific synthesis of the literature within the Great Plains and Midwestern USA.

## Graphical abstract

This systematic review describes the literature regarding ring‐necked pheasant habitat needs throughout the year in central North America. Pheasant's require different resources during each of the three primary life‐history stages—breeding/nesting, brood‐rearing, and overwintering. Many studies investigate only one or two of these seasons; this review synthesizes those studies to identify seasonal trends and gaps in our knowledge of pheasant habitat.

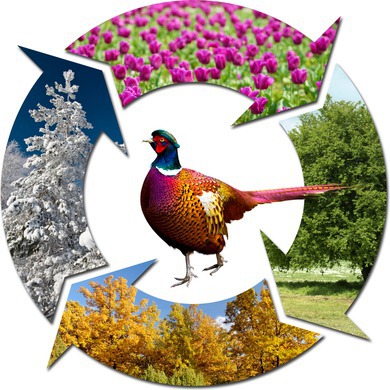

## 1 Introduction

Ring‐necked pheasant (*Phasianus colcichus*; hereafter, pheasant) populations across North America have been in decline for over 60 years (Dahlgren 1988; Sauer et al. 2013). First introduced to North America in the nineteenth century, pheasants have adapted to a wide array of habitat types, but have found particular success among the agricultural landscapes of the central United States (Dahlgren 1988). Although often regarded as a grassland species in North America, ring‐necked pheasants are more accurately described as habitat generalists (Liu et al. 2020), or shrub/scrub species. In their native range of Asia and naturalized range in Europe, they are thought of as a woodland or shrubland species and are frequently found roosting in woodlands with a dense shrub layer (Ashoori et al. 2018; Chiatante and Meriggi 2022; Draycott et al. 2005, 2008; Li et al. 2009; Robertson 1997). When introduced to central North America, where native woodlands were scarce, they became more strongly associated with grasslands and wetlands that were able to provide sufficient cover and structure (Applegate et al. 2003; Clark and Bogenschutz 1999; Hiller et al. 2015). As is the case with many generalist species, pheasant habitat use is landscape dependent, making it necessary to examine the unique ecological and environmental factors and interactions that influence pheasant habitat use across regions in order to inform management.

The ability to exploit many different land cover types enabled pheasants to thrive in the diverse mosaics of row crops, small grains, pasture, grassland, wetland, and tree stands characteristic of central North America (Nielson et al. 2008; Smith et al. 1999). However, advances in agricultural mechanization in the 1950s and the subsequent intensification of cropping systems resulted in once‐diverse agricultural landscapes transitioning to domination by corn ( *Zea mays* ) and soybean ( *Glycine max* ) monocultures, along with conversion of substantial proportions of remaining wetlands, pastures, fencerows, and grasslands to row crops (Hiller et al. 2009; Pretty and Bharucha 2014). These land use changes were accompanied by gradual declines in pheasant populations (Powell 2015; Warner et al. 1984).

**FIGURE 1:**
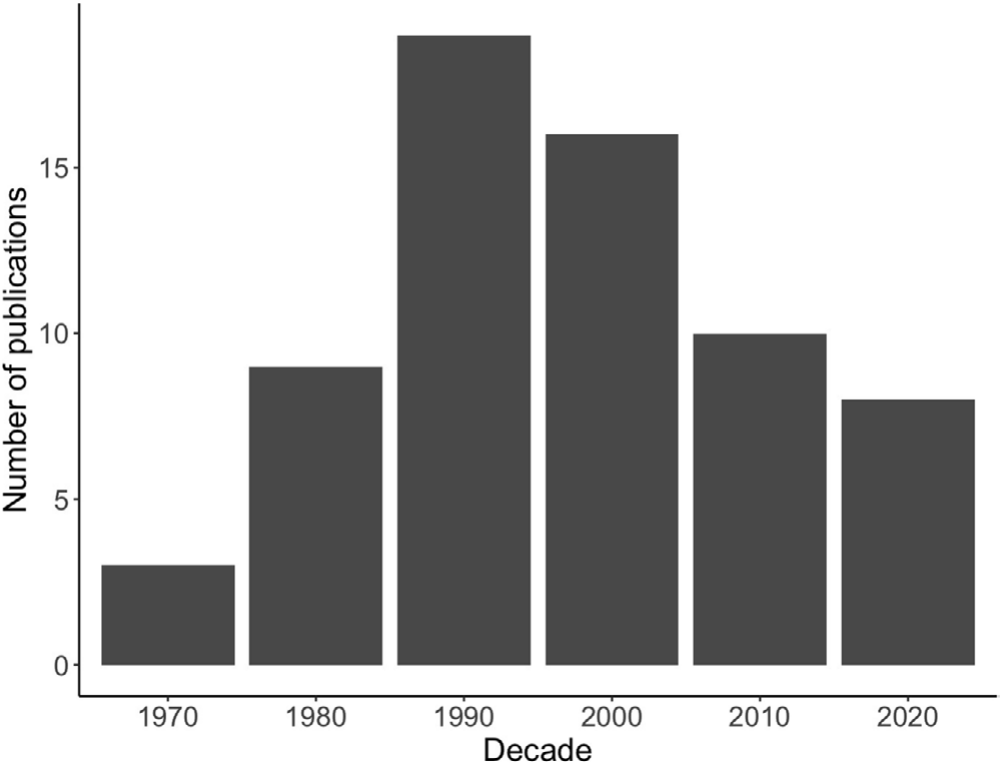
Decadal counts of peer‐reviewed publications on pheasant habitat, from 1970 to 2023, with the most recent decade covering 2020–2023.

Declining pheasant populations are concerning for both recreationists and ecologists. Pheasant hunting is an important industry in many of the midwestern states such as South Dakota, where it is estimated that hunting generates up to $223 million each year (Errington and Gewertz 2015), and Nebraska, where it generates approximately $32 million each year (Midwest Pheasant Study Group 2013), much of which goes to rural communities. Although they are an introduced species, there are also ecological implications of pheasant declines. Some ecologists have referred to pheasants as an indicator of ecosystem health in agriculturally dominated landscapes due to their historically strong relationships with these ecosystems (Hallett et al. 1988), leading to concerns about what decreasing populations indicate about broader biodiversity. As these concerns grew, so did the number of ecological studies with a focus on relationships between habitat management and pheasant survival, resource use, movement, and abundance (Figure 1).

Many studies on pheasant habitat focus on individual seasons or life stages—breeding/nesting (hereafter; breeding), brood‐rearing, or overwintering—and subsequently make management recommendations based on the findings. However, managing for life stages in isolation may not achieve population‐level conservation goals if the needs of other stages are not considered. This is because pheasant habitat needs change throughout the year based on their life history. Pheasants have a unique social system known as territorial harem defense polygyny which dictates much of their life history (Ridley and Hill 1987). Overwinter, pheasants tend to congregate in groups, often segregated by sex (Collias and Taber 1951). Each spring, males compete for territory while females move in groups to select a male with whom they will mate for the season in exchange for protection from predators and other males (Ridley and Hill 1987). Females generally nest near the edges of selected male territories and will renest multiple times if nests or broods are lost (Dumke and Pils 1979). After the hatching of chicks, females divert their efforts from breeding and nesting to brood‐rearing before moving back to their winter home ranges. Each of these stages creates distinct challenges to survival which dictate how pheasants utilize the resources available to them, including changes in habitat selection.

Despite decades of research on pheasant habitat in central North America, there remain gaps in our understanding of their changing habitat needs throughout the year. Given that previous studies have provided key insights on specific seasons or life‐history stages, such as overwintering, breeding, or brood‐rearing, there is an opportunity for a robust synthesis that spans seasons and addresses the interplay between them. This review synthesizes the findings of peer‐reviewed scientific literature in central North America, specifically the Great Plains and Midwest regions of the United States, in order to increase understanding of changing pheasant habitat use throughout the year and inform conservation. Through synthesis of findings across seasons and geography, our study aims to contribute to the conservation and management of pheasant populations and the agricultural landscapes they inhabit. Our review emphasizes patterns in reported habitat requirements over multiple seasons, and in doing so, provides a foundation for further research and management amidst ongoing environmental and landscape change.

## 2 Methods

The geographic scope of this review was the Great Plains and Midwest Region of the United States due to their relative similarities in land use, topography, and climate, as well as the history of supporting pheasant populations. To address the variability and subjectivity in defining the boundaries of these regions, we used the Great Plains Region outlined by Lavin et al. (2011) and the Midwest Region definition provided by National Geographic (O'Conner 2024). Notably, these two regions overlap, with portions of North Dakota, South Dakota, Nebraska, and Kansas included in both. The temporal extent of the review was from 1970 to 2023, which captures the period in which pheasant habitat became a priority in ecological research due to population declines.

A systematic literature search was conducted according to the PRISMA guidelines for ecology and evolution (O'Dea et al. 2021) using the Web of Science “all databases” search function. Search terms and Boolean operators used are reported in Table 1. Subsequently, search results were refined to include only peer‐reviewed articles originating from the United States. This yielded a total of 174 articles, which were then subjected to manual review by two independent reviewers, the lead authors of this paper, to determine if they met inclusion criteria. Papers were included if any portion of the study was conducted within the study area described above. Additional inclusion criteria were: (1) the study must appear in a peer‐reviewed journal; (2) must present original research on pheasant habitat, resource use, or survival directly related to habitat; (3) must study wild, in‐situ pheasant populations; (4) no genetic studies were included; (5) no diet analyses were included; (6) multi‐species studies were included only if they reported species‐specific results; and (7) biogeographic studies were included only if they evaluated habitat or resource use as a driver of changes in range or distribution. Books, reports, theses, dissertations, gray literature, magazines, newsletters, fact sheets, conferences, seminar or symposium proceedings, assessment projects, reviews, guides, and bibliographies were excluded. Studies using a mix of wild and captive or translocated pheasants were excluded if the majority of pheasants were captive or translocated. After this manual selection, 65 papers remained and were used in the analysis (see Appendix A). The abstract, methods, and results of each study were thoroughly reviewed and summarized in Microsoft Excel, and the relevant information was imported into R (R Core Team 2024) and interpreted for trends, differences, and commonalities.

**TABLE 1:** Search terms used on the Web of Science databases. Search was conducted using the “All Databases” search option.

Search number	Location of search	Keywords	Search number combination
#1	Title	Pheasant OR “ *Phasianus colchicus* ”	
#2	Abstract		
#3	Title	Habitat OR mesohabitat OR macrohabitat OR microhabitat OR “food plots” OR agriculture OR cover‐type OR “nest site” OR “land use” OR “resource selection” OR “resource use” OR “cover preferences”	
#4	Abstract		
#5			#1 OR #2
#6			#3 OR #4
#7			#5 AND #6

Studies were categorized based on the season(s) during which data were collected. If a study collected data during multiple seasons and used that data to draw broad conclusions about habitat use, resource selection, or survival throughout the year, then the study was categorized as “annual”. For example, Warner and Joselyn (1986) utilized data from spring crow counts, winter helicopter counts, summer nest counts, and fall/winter interviews with hunters to investigate population responses to management over the course of several years. Alternatively, studies that collected data during multiple seasons and analyzed those results separately, reporting results for each season, were classified by the seasonal categories for which they had results. For example, Whiteside and Guthery (1983) collected radio telemetry data year‐round and reported results for movements and habitat use during the winter, spring, summer, and fall seasons individually over the course of a year. There is some overlap between the breeding season and the brood‐rearing season because some pheasant hens will continue to breed and nest (or renest) throughout the summer while others have entered the brood‐rearing stage (Dumke and Pils 1979). We classified breeding/nesting season studies as those that collected data on male pheasants from March through August, or those that specifically referred to nesting females, nest site selection, or nest success. If a paper specified that they studied hens with broods, these were classified as brood‐rearing studies. This differentiation was made with the assumption that habitat needs change based on life stage rather than the month of the year, meaning that if a hen is still nesting in July, her habitat needs will be different from a hen who has successfully nested and is raising a brood at that point. Seven studies reported results for both the breeding season and the brood‐rearing season independently, so they were included in results for both categories.

Land cover types were hierarchically classified in order to standardize the results of each study for quantitative analysis. Grassland includes both private and public grasslands, including United States Department of Agriculture—Conservation Reserve Program (CRP) fields; woody cover includes shrublands, woodlands and shelterbelts; wetland includes herbaceous and woody wetlands; linear features include roadsides, fencerows, waterways, and railroad rights‐of‐way; and agricultural grassland includes hay fields, alfalfa, and pastures. Wheat and wheat stubble were incorporated into the small grains category. Percentage or abundance of litter and forbs were included as grassland structural components due to their relevance in the literature to microhabitat and nest site selection (Matthews et al. 2012a). For each analyzed paper, each land cover category was designated as “positive”, “negative”, “variable”, “no effect found”, or “not included in analysis”; where, positive/negative are defined as having a positive or negative relationship with pheasant abundance, occupancy, habitat selection, survival, or nest/brood success in at least one spatial scale. A land cover category was labeled as “no effect found” if it was measured during the study but did not appear in the top model or no statistically significant relationship was found. A land cover category was labeled as “variable” if the direction of the relationship changed based on scale, study area, or some other studied predictor (e.g., row crops showed a positive relationship with pheasant abundance at the home range scale but a negative relationship at the landscape scale).

## 3 Results

### 3.1 Annual Studies

**FIGURE 2:**
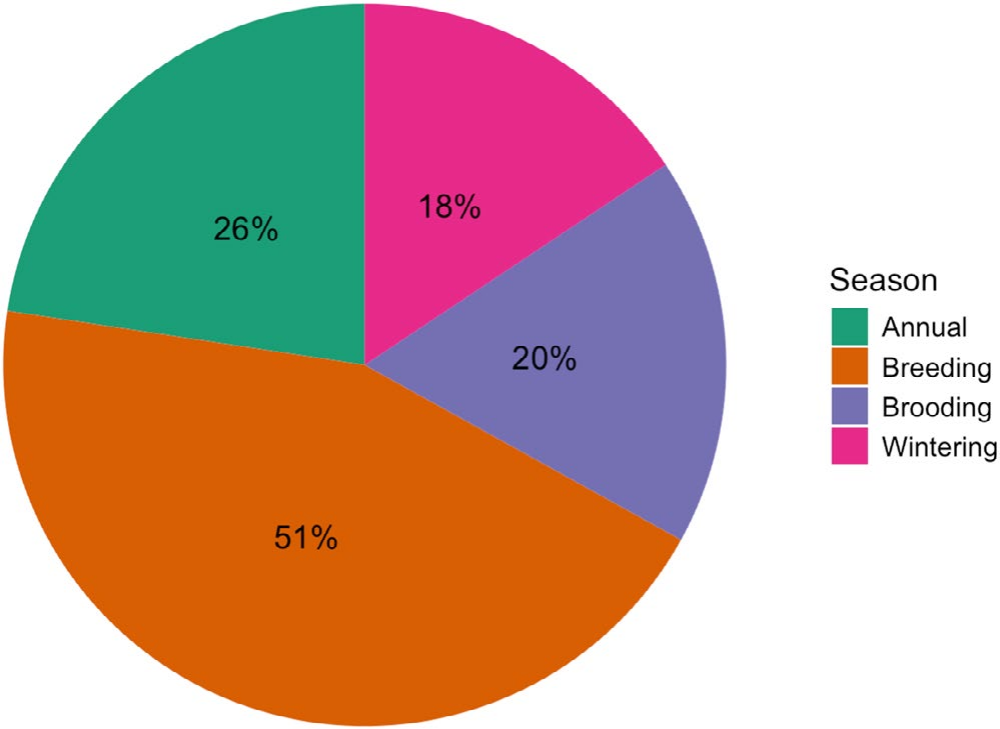
Percentage of papers reporting results for each of the key life stages for pheasants.

Out of 65 papers analyzed, 17 (26%) reported results on an annual, not seasonal, basis (Figure 2). These studies either (a) used data collected during one life stage, such as spring crow counts, to draw broad conclusions about pheasant habitat (e.g., Jorgensen et al. 2014; Stuber et al. 2017) or (b) synthesized results collected at multiple points throughout the year to identify overall trends (e.g., Emmet et al. 2023; Giudice and Haroldson 2007).

Eleven studies included in the “annual” category included a grassland variable, of which five (46%) reported a positive relationship, while three (27%) found no significant relationship, and three (27%) found that the direction of the relationship was variable, or changed at some scale or threshold of grassland coverage (Table 2). Eleven of these studies included row crops in their analysis, with one (9%) reporting a positive relationship, two (18%) finding a negative relationship, six (55%) finding no significant relationship, and two (18%) finding that the direction of the relationship was variable. Small grains also appeared in 11 studies, of which six (55%) reported a positive relationship, three (27%) found no relationship, and two (18%) found that the relationship varied. These three variables were the most commonly studied among papers categorized as “annual”, followed by agricultural grasslands [*n* = 7; positive = 2 (29%), no relationship = 5 (71%)] and woodlands [*n* = 7; negative = 4 (57%), no relationship = 3 (43%)].

**TABLE 2:** Results of the literature review for annual studies that did not report results for individual seasons.

Season	Variable	Number of studies included	Relationship	Number (%)
Annual (*n* = 17)	Grass/CRP	11	Positive	5 (46%)
			Negative	0
			No relationship	3 (27%)
			Varied	3 (27%)
	Linear grassy habitats	3	Positive	2 (67%)
			Negative	0
			No relationship	1 (33%)
			Varied	0
	Vegetation height	2	Positive	1 (50%)
			Negative	0
			No relationship	1 (50%)
			Varied	0
	Forb content	1	Positive	0
			Negative	0
			No relationship	1 (100%)
			Varied	0
	Litter content	1	Positive	0
			Negative	0
			No relationship	1 (100%)
			Varied	0
	VOR	1	Positive	0
			Negative	0
			No relationship	1 (100%)
			Varied	0
	Row crops	11	Positive	1 (9%)
			Negative	2 (18%)
			No relationship	6 (55%)
			Varied	2 (18%)
	Small grains	11	Positive	6 (55%)
			Negative	0
			No relationship	3 (27%)
			Varied	2 (18%)
	Hay/pasture	7	Positive	2 (29%)
			Negative	0
			No relationship	5 (71%)
			Varied	0

	Disturbance	1	Positive	0
			Negative	0
			No relationship	0
			Varied	5 (100%)
	Wetlands	5	Positive	2 (40%)
			Negative	0
			No relationship	3 (60%)
			Varied	0
	Woodlands	7	Positive	0
			Negative	4 (57%)
			No relationship	3 (43%)
Varied			0

### 3.2 Breeding and Nesting Studies

Thirty‐two studies investigated pheasant habitat during the spring breeding and nesting season (Table 3). During this season, the literature emphasized the importance of grass and grasslands as pheasant habitat, particularly for nesting. Out of 26 papers that included a grass and/or CRP variable, 20 (77%) found a positive relationship, one (4%) found a negative relationship, one (4%) found no relationship, and three (12%) found that the relationship varied. Linear grassy features [*n* = 17; positive = 4 (24%), negative = 4 (24%), no relationship = 9 (53%)] and agricultural grasslands [*n* = 15; positive = 4 (27%), negative = 3 (20%), no relationship = 7 (47%), varied = 1 (7%)] also appeared in the literature, with mixed results.

**TABLE 3:** Results of literature review for studies during the breeding and nesting season.

Season	Variable	Number of studies included	Relationship	Number (%)
Breeding/nesting (*n* = 32)	Grass/CRP	26	Positive	20 (76%)
			Negative	1 (4%)
			No relationship	1 (4%)
			Varied	3 (12%)
	Linear grassy habitats	17	Positive	4 (24%)
			Negative	4 (24%)
			No relationship	9 (53%)
			Varied	0
	VOR	10	Positive	8 (80%)
			Negative	0
			No relationship	2 (20%)
			Varied	0
	Vegetation height	6	Positive	6 (100%)
			Negative	0
			No relationship	0
			Varied	0
	Forb content	9	Positive	4 (45%)
			Negative	0
			No relationship	5 (55%)
			Varied	0
	Litter content	5	Positive	6 (100%)
			Negative	0
			No relationship	0
			Varied	0
	Row crops	15	Positive	3 (20%)
			Negative	10 (67%)
			No relationship	2 (13%)
			Varied	0
	Small grains	12	Positive	7 (58%)
			Negative	2 (17%)
			No relationship	2 (17%)
			Varied	0
	Hay/pasture	15	Positive	4 (27%)
			Negative	3 (20%)
			No relationship	7 (47%)
			Varied	1 (7%)

	Disturbance	7	Positive	5 (71%)
			Negative	0
			No relationship	2 (29%)
			Varied	0
	Edge metrics	5	Positive	3 (60%)
			Negative	2 (40%)
			No relationship	0
			Varied	0
	Patch size	7	Positive	5 (71%)
			Negative	0
			No relationship	2 (29%)
			Varied	0
	Wetlands	7	Positive	4 (57%)
			Negative	1 (14%)
			No relationship	2 (29%)
			Varied	0
	Woodlands	11	Positive	4 (37%)
			Negative	1 (9%)
			No relationship	5 (45%)
Varied			1 (9%)

We found 14 studies that highlighted the importance of grassland structure including Visual Obstruction Readings (VOR), vegetation height, litter composition and forb content. VOR was the most commonly studied of these (*n* = 10), with seven studies (70%) reporting a significant, positive correlation between this variable and pheasants during the breeding season. Forb (also commonly referred to as weed) abundance in grasslands appeared in nine studies, of which four (45%) reported a positive relationship and five (55%) reported no relationship. All studies that included litter content (*n* = 5) and vegetation height (*n* = 6) reported significant positive relationships (100%). Notably, all studies that reported relationships between grassland structure and pheasants were focused on nest success, nest site selection, and/or recruitment, which demonstrates the importance of late successional stage grasslands with tall vegetation, high litter composition and high VOR for nesting females.

Reported impacts of agriculture on pheasants during the breeding and nesting season varied according to the thematic resolution of the landcover data being used (Baldissara et al. 2025). For example, some studies grouped all agricultural lands into one category, others divided them into crop and non‐crop classifications, while many included individual crop types as separate classes. Three studies grouped all agricultural classes into one variable, with two (67%) finding a positive relationship, and one (33%) finding a negative relationship. Out of studies that used finer thematic resolution in their classifications, the most commonly included category was row crops (*n* = 15). Of these, 10 (67%) found a negative relationship, three (20%) found a positive relationship, and two (13%) found no relationship. Twelve papers included small grains, of which seven (58%) reported a positive relationship, two (17%) reported a negative relationship, and two (17%) reported no relationship.

The effects of habitat configuration are challenging to assess because of the diversity of metrics used to quantify it. However, there were some commonalities, such as the effects of the amount of edge habitat and the size of habitat patches (generally referring to grassland patches). Five studies during the breeding and nesting season incorporated edge metrics, but the findings were split [positive = 3 (60%), negative = 2 (40%)]. Patch size appeared in seven studies, with five (71%) reporting a positive relationship and two (29%) reporting no relationship.

### 3.3 Brood‐Rearing Studies

Thirteen studies examined pheasant habitat during the brood‐rearing season, with six focusing exclusively on broods and seven investigating multiple seasons but reporting seasonal results independently (Table 4). Grasslands were once again emphasized during this season (*n* = 9). Six (67%) studies that included a grassland variable found a significant, positive relationship with broods (e.g., presence, abundance, success), while one (11%) found no relationship and two (22%) found that the relationship varied. The relationship with grassland structure is not as pronounced during the brooding season as in the breeding season; however, five papers included at least one variable representing vegetation structure (litter, VOR, vegetation height, or forb abundance), all of which reported positive relationships. Once again, linear grassy features [*n* = 5; positive = 3 (60%), negative = 1 (20%), no relationship = 1 (20%)] and agricultural grasslands [*n* = 6; positive = 3 (50%), no relationship = 3 (50%)] showed mixed results.

**TABLE 4:** Results of the literature review for studies during the brood‐rearing season.

Season	Variable	Number of studies included	Relationship	Number (%)
Brood rearing (*n* = 13)	Grass/CRP	9	Positive	6 (67%)
			Negative	0
			No relationship	1 (11%)
			Varied	2 (22%)
	Linear grassy habitats	5	Positive	3 (60%)
			Negative	1 (20%)
			No relationship	1 (20%)
			Varied	0
	VOR	2	Positive	2 (100%)
			Negative	0
			No relationship	0
			Varied	0
	Vegetation height	1	Positive	1 (100%)
			Negative	0
			No relationship	0
			Varied	0
	Forb content	2	Positive	2 (100%)
			Negative	0
			No relationship	0
			Varied	0
	Litter content	1	Positive	1 (100%)
			Negative	0
			No relationship	0
			Varied	0
	Row crops	8	Positive	1 (11%)
			Negative	4 (50%)
			No relationship	3 (33%)
			Varied	0
	Small grains	6	Positive	4 (60%)
			Negative	0
			No relationship	2 (40%)
			Varied	0
	Hay/pasture	6	Positive	3 (50%)
			Negative	0
			No relationship	3 (50%)
			Varied	0

	Disturbance	1	Positive	0
			Negative	1 (100%)
			No relationship	0
			Varied	0
	Wetlands	3	Positive	1 (33%)
			Negative	0
			No relationship	2 (67%)
			Varied	0
	Woodlands	3	Positive	1 (33%)
			Negative	0
			No relationship	2 (67%)
Varied			

Row crops were included in eight studies, four (50%) of which reported a negative relationship, one (11%) a positive relationship, and three (33%) no relationship. Small grains appeared in six studies with three (50%) finding a positive relationship and three (50%) finding no relationship.

### 3.4 Overwintering Studies

Thirteen studies investigated pheasant habitat during the winter season (Table 5). Grasslands were the most frequently studied land cover type, appearing in nine studies, eight (89%) of which found a significant, positive relationship, while one (11%) found no relationship. Grassland structure was not commonly studied during this season; however, all studies that included vegetation height (*n* = 3, 100%) and forb content (*n* = 2, 100%) found a positive relationship. Linear features (*n* = 1) and agricultural grasslands (*n* = 3) were found to have negative relationships with pheasants during the winter, albeit in a relatively small number of studies.

**TABLE 5:** Results of the literature review for studies during the winter season.

Season	Variable	Number of studies included	Relationship	Number (%)
Overwintering (*n* = 13)	Grass/CRP	9	Positive	8 (89%)
			Negative	0
			No relationship	1 (11%)
			Varied	0
	Linear grassy habitats	1	Positive	0
			Negative	1 (100%)
			No relationship	0
			Varied	0
	Vegetation height	3	Positive	3 (100%)
			Negative	0
			No relationship	0
			Varied	0
	Forb content	2	Positive	1 (100%)
			Negative	0
			No relationship	0
			Varied	0
	Row crops	6	Positive	1 (17%)
			Negative	3 (50%)
			No relationship	0
			Varied	2 (33%)
	Small grains	3	Positive	3 (100%)
			Negative	0
			No relationship	0
			Varied	0
	Food plots	6	Positive	5 (83%)
			Negative	0
			No relationship	1 (17%)
			Varied	0
	Hay/pasture	3	Positive	0
			Negative	3 (100%)
			No relationship	0
			Varied	0
	Edge metrics	2	Positive	1 (50%)
			Negative	1 (50%)
			No relationship	0
			Varied	0

	Patch size	2	Positive	2 (100%)
			Negative	0
			No relationship	0
			Varied	0
	Wetlands	8	Positive	6 (75%)
			Negative	0
			No relationship	2 (25%)
			Varied	0
	Woodlands	8	Positive	2 (25%)
			Negative	0
			No relationship	5 (63%)
Varied			1 (12%)

Pheasant relationships with croplands during the winter are complex, as these fields are a source of food but offer little in the way of shelter after harvest. Out of six studies that included row crops, three (50%) found a negative relationship, one (17%) a positive relationship, and two (33%) found that the relationship changed direction during the study. Small grains, including wheat and wheat stubble, however, were found to have a positive relationship in all three (100%) papers in which they were included. Two of these specifically focused on wheat stubble as winter habitat, which offers the additional benefit of providing foraging opportunities and some level of shelter depending on the stubble height (Rodgers 1999, 2002).

Several land cover categories appeared more prominently in the winter literature than in any other season. Food plots—stands of corn left standing over winter specifically to provide food for pheasants and other game birds—appeared in six papers, five (83%) of which found a positive relationship with pheasants. Woodlands (*n* = 8) and wetlands (*n* = 8) also appear more frequently in the winter literature due to the hypothesis that pheasants utilize these cover types during harsh weather. This was supported for wetlands [positive = 6 (75%), no relationship = 2 (25%)], but not for woodlands [positive = 2 (25%), no relationship = 5 (63%), varied = 1 (12%)].

## 4 Discussion

Past pheasant habitat research has built a substantial body of literature from which researchers and managers can learn, but many studies have primarily focused on a single season or life stage. Pheasants have different habitat needs throughout the year, particularly in the three key ecological stages—breeding, brood‐rearing, and overwintering. With pheasant populations continuing to decline in North America, it is crucial to approach habitat management from a year‐round perspective. Here, we have synthesized the literature on pheasant habitat needs throughout the year in central North America and have found several key commonalities. This type of review provides important insights for those looking to make decisions regarding pheasant management and conservation across seasons.

It is clear from the literature that pheasant habitat is highly context‐and scale‐dependent (Amirkhiz et al. 2023; Stuber et al. 2017). Unlike many grassland obligate species occupying the same habitats, pheasants rely on a mosaic of grassland, agriculture, and other habitat types for resources (Hagen et al. 2007; Haroldson et al. 2007). Among studies that reported results on an annual basis, rather than during a particular season, only 45% that included grasslands in their analysis reported a significant, positive relationship on pheasant abundance or resource selection, while the rest found either no relationship or reported that the relationship became negative at some particular scale or threshold of grassland coverage (e.g., Haroldson et al. 2007). When considering annual studies that included row‐crops, we found that 55% found no relationship and 9% found a positive relationship. The variation of these results suggests that there is nuance to these relationships that is not fully captured within the temporal scale of these studies. Additionally, many of these relationships are likely non‐linear, which is difficult to capture in all studies based on differences in data collection and analysis methods. These nuances become clearer when studies are broken down by life stage.

While our results demonstrate the importance of access to different habitat types throughout the year, it is not clear how the configuration of these features influences pheasant resource use and abundance. While several papers included at least one metric of configuration (e.g., Simonsen and Fontaine 2016), it was difficult to identify commonalities between them due to the wide variety of metrics used. The most commonly used metrics were edge amount (Gatti and Schneider 2015; Hagen et al. 2007; Solem and Runia 2022) and patch size (Clark et al. 1999; Gatti et al. 1989; Riley 1992). Solem and Runia (2022) and Hagen et al. (2007) reported a positive relationship between pheasants and edge amount, while Gatti and Schneider (2015) found a negative relationship. Patch size was included in 10 studies, seven of which found that larger patch sizes were related to increased nest abundance, nest success, or pheasant abundance (Clark et al. 1999; Clark and Bogenschutz 1999; Gatti et al. 1989; Gatti and Schneider 2015; Riley 1992; Schmitz and Clark 1999; Solem and Runia 2022). However, not all studies agreed on the effect of patch size. While Clark and Bogenschutz (1999) found that nests in “block habitats” were more successful than those in linear habitats, Warner (1994) reported that nests found in roadsides and other linear habitats had as high, or higher, success than those in large fields. These inconsistencies speak to the complexity of the ecological relationships between landscape composition and configuration, and how each of those influences pheasants within the landscape context.

### 4.1 Nesting and Breeding Season

The spring breeding and nesting season was the most commonly studied season in our review (*n* = 32; 49%), and there are both ecological and practical reasons for researchers to target this season. Nesting habitat is hypothesized to be the most limiting factor affecting population declines (Robertson 1996) and increasing nest and brood survival is a priority for their conservation (Matthews et al. 2012a; Pauly et al. 2018; Robertson 1996). The spring breeding season is also the time when male pheasants are out crowing at regular intervals, making it the ideal time to conduct crow counts—a commonly used method to estimate male abundance (Kimball 1949). As a result, studies examining pheasant abundance during this season primarily focus on males, while those studying nest site selection and success almost exclusively focus on females, despite the role that male territory selection likely plays in nesting ecology (Clark et al. 1999; Dumke and Pils 1979). In fact, only two studies used radio telemetry to study male habitat use during the spring (Leif 2005; Whiteside and Guthery 1983), compared to 13 radio telemetry studies on females during the same season. We see this as an important knowledge gap in the pheasant literature in North America. Research has shown that females nest in or near the territory of their selected male (Clark et al. 1999; Dumke and Pils 1979; Ridley and Hill 1987), making male territory selection an important factor in later nesting and brood‐rearing ranges.

Nineteen studies during the nesting season specifically investigated nest site selection or nest success relative to nest location. The results of these highlighted the importance of late successional grasslands with tall vegetation, high VOR, and high litter content. This marked preference for tall, dense vegetation coverage may explain some of the variation seen in the review regarding alternative grassland classifications such as linear grassy features (roadsides, fencerows, etc.), hay fields, and pastures. Several papers found that pheasants utilize these cover types for nesting (Camp and Best 1994; Dumke and Pils 1979; Hanson and Progulske 1973; Paruk 1990; Warner and Joselyn 1986); however, nest attempts were not as successful as those in block grassland cover (Hanson and Progulske 1973; Patterson and Best 1996). While structurally many agricultural grasslands such as hay fields and pastures are suitable for nesting, frequent disturbance by mowing or grazing leads to high rates of nest failure (Hanson and Progulske 1973; Patterson and Best 1996). In the case of linear features, particularly roadsides, management and surrounding landscape are key to whether or not nesting is likely to be successful. Warner (1984) found that nests in roadsides managed for wildlife with no mechanical disturbance were as successful as those in surrounding block habitats and more so than in nearby hay fields, which demonstrates the potential value of marginal and strip grassland cover if managed with avian nesting in mind.

### 4.2 Brood‐Rearing Season

Pheasant brood habitat is considered to be more open in structure than nesting areas, with a tall overhead canopy and an understory containing bare ground for ease of movement. We found only two studies that directly tested the effects of grassland structural components on broods, but these studies did confirm that hens with broods selected and were more successful in managed grasslands that had high forb abundance—thus a more open understory structure than is provided by dense grasses—and greater VOR (Matthews et al. 2012a, 2012b). Additionally, Eggebo et al. (2003) found greater brood use in cool season grasses that were one or more years post‐establishment, and also found that these fields had greater vegetation structure (i.e., taller and denser vegetation). Previous studies that did not fall within the scope of this review have also shown that chick foraging is more efficient in grasslands with greater amounts of bare ground, and that fields with higher forb abundance have higher invertebrate diversity (Doxon and Carroll 2007, 2010). This same structure can also be found in many weedy wheat and small grain fields, making these ideal foraging habitats for broods when not treated with pesticides and herbicides (Warner 1984). Four out of six papers that explored pheasant brood relationships with small grains found a positive relationship, suggesting that this may be an important source of habitat in agricultural regions. In recent decades, however, small grains have declined significantly across the United States, with both wheat and oats declining by more than 50% in average annual acres planted between 1970 and 2020 (United States Department of Agriculture (USDA) 2025). This change, combined with the adoption of so‐called 'clean' farming practices involving the use of insecticides and herbicides, has led to the loss of this habitat in most locations that set‐aside programs such as CRP cannot adequately compensate for (Rodgers 1999; Warner et al. 1999).

Row crops were the second most studied land cover type (*n* = 8), after grasslands. Half of the papers (*n* = 4) that included row crops found them to be negatively associated with broods, while three (33%) found no relationship. One study, Hanson and Progulske (1973), reported that hens with broods did frequently use corn fields in their study conducted from 1969 to 1970. However, similar to the case of small grains and wheat fields, clean farming practices and intensification of farming systems to increase yields have reduced the value of crop fields as habitat for pheasant chicks (Warner et al. 1999). More recent studies found that broods were rarely found in crop fields in Iowa despite their prevalence (Riley et al. 1998), and Matthews et al. (2012a) reported that brood survival decreased as time spent in crop fields increased. A similar trend was found with gray partridge ( *Perdix perdix* ) in Great Britain, where chick survival rates dropped 17% after the introduction of herbicides (Potts and Aebischer 1995).

### 4.3 Overwintering

Thirteen studies investigated pheasant habitat use during the winter months. The results of these studies agree that grasslands are an important habitat feature, although the relationship with vegetation structure, including Visual Obstruction Readings (VOR), vegetation height, litter composition, and forb content, that appeared in the previous seasons is not as apparent here. Vegetation height was the only structural feature to appear more than once in the literature, and all three studies that included vegetation height found it to have a significant, positive relationship with pheasant habitat use and survival. Alternative grasslands such as roadsides, hay fields, and pastures were reported to be universally avoided within the winter literature (Gabbert et al. 1999; Gatti et al. 1989; Homan et al. 2000), possibly due to fall haying and grazing leaving them without adequate winter cover.

Food plots are a land cover category that is unique to the winter literature. The planting of food plots, stands of corn or grains that are left unharvested over winter specifically to provide food for pheasants and other game birds, is a practice that several studies have shown to be valuable (Gabbert et al. 1999; Gatti et al. 1989; Kauth et al. 2022; Larsen et al. 1994; Riley 1992). Unlike harvested crop fields, which are also used for winter foraging, food plots may offer shelter in addition to food (e.g., corn stalks left standing through the winter). Riley (1992) reported that food plots over four hectares in size were most heavily used by pheasants, and Larsen et al. (1994) found that food plots near cattail wetlands produced the highest number of birds. Provisioning food sources near high‐quality winter habitat is a common practice in European pheasant management (Sánchez‐García et al. 2015), where planting stands of cereal grains or placing hoppers of grain near winter cover has led to increased winter survival (Draycott et al. 2002). This was also found in our review by Gabbert et al. (2001), who reported that female pheasants with food plots within their home range had a significantly higher winter survival than those without.

During the winter season, wetlands and woodlands both become more prominent across the literature, appearing in eight studies each. Three quarters (*n* = 6; 75%) of studies that included wetlands found them to have an important, positive relationship with pheasant habitat use and survival. In some locations, cattail wetlands may represent the only tall standing cover on the landscape that is dense enough to stand up to strong winds and snow (Kauth et al. 2022; Schneider 1985). Several studies found that as winter weather worsened, pheasants used wetlands more frequently (Homan et al. 2000; Perkins et al. 1997). Similarly, woodlands received greater use during harsh winter weather than any other time of year. While most (63%) studies that included woodlands did not find a significant relationship, several reported that woodlands, including shrublands, forests, pine groves, and shelterbelts, became important during periods of heavy snowfall when weather‐related mortality was highest (Gabbert et al. 1999; Homan et al. 2000; Kauth et al. 2022; Perkins et al. 1997). Gatti et al. (1989) found that all pheasants included in their study included some form of woody cover in their winter home range. Haroldson et al. (2007) did not find a relationship between pheasant abundance and winter cover, defined as a combination of undisturbed grass, wetlands, and food plots, but acknowledged that their study was conducted under mild winter conditions. Increased winter mortality is directly linked to the severity of weather conditions, with worse conditions leading to higher mortality from both exposure and predation (Dumke and Pils 1979; Gabbert et al. 1999; Perkins et al. 1997). This highlights the importance of scale in the matter of winter habitat. Pheasant home ranges are generally smallest in winter (Gatti et al. 1989; Whiteside and Guthery 1983), and larger home ranges and increased movement decrease winter survival in some populations (Gatti et al. 1989). When severe winter weather does occur, it is crucial for pheasant survival that birds be able to access tall, dense standing cover such as cattail wetlands, shrubs, and woodlands within their winter ranges. Additionally, increasing food plot size and density near these cover types may lead to increased survival in locations with harsh winter weather where pheasant mortality is likely to be greatest (Gabbert et al. 2001; Perkins et al. 1997).

## 5 Conclusion

This review of the ring‐necked pheasant habitat literature aims to contribute to the conservation and management of pheasant populations within agricultural landscapes, and to provide a foundation for further research by identifying trends and gaps in our knowledge of pheasant habitat ecology. Our results confirm that pheasants require a variety of habitat types throughout the year, including mixed agricultural lands and undisturbed vegetation in varying stages of succession. Female pheasants select for tall, late successional grasslands with high litter content for nesting, while broods have higher success in land cover with high forb content and a more open structure at ground level. Strategies such as staggering mid‐contract management practices in neighboring CRP fields may help provide these conditions in close proximity. Additionally, access to tall standing vegetation such as cattail wetlands is important for winter survival, particularly in harsh winter weather. Considering differences in seasonal resource needs and planning restoration and conservation actions according to the greater landscape context may be a key component in reducing population declines in agriculturally dominated landscapes.

## Author Contributions

**Allison J. Barg:** conceptualization (equal), data curation (equal), investigation (lead), methodology (equal), writing – original draft (lead). **Megan Baldissara:** conceptualization (equal), data curation (equal), methodology (equal). **Daniel R. Uden:** conceptualization (equal), methodology (equal), supervision (supporting), writing – review and editing (equal). **John P. Carroll:** investigation (supporting), supervision (supporting), writing – review and editing (equal). **Andrew Little:** conceptualization (equal), funding acquisition (lead), methodology (equal), supervision (lead), writing – review and editing (equal).

## Conflicts of Interest

The authors declare no conflicts of interest.

## Data Availability

The data that support the findings of this study are openly available in DRYAD at https://doi.org/10.5061/dryad.j3tx95xr4.
